# Comparative Efficacy and Safety of Self-Expandable vs. Balloon-Expandable Stent Grafts in Visceral Artery Aneurysm Management

**DOI:** 10.3390/diagnostics14151695

**Published:** 2024-08-05

**Authors:** Reza Talaie, Pooya Torkian, Anthony Spano, Alireza Mahjoubnia, Siobhan M. Flanagan, Michael Rosenberg, Jian Lin, Jafar Golzarian, Preshant Shrestha

**Affiliations:** 1 Vascular and Interventional Radiology, Department of Radiology, University of Minnesota, Minneapolis, MN 55455, USA; 2 Department of Mechanical and Aerospace Engineering, University of Missouri, Columbia, MO 65211, USA

**Keywords:** visceral artery aneurysm, self-expandable stent, balloon-expandable stent, endoleak

## Abstract

Purpose: This study assesses the efficacy and safety of self-expandable (SE) versus balloon-expandable (BE) stent grafts for managing visceral artery aneurysms (VAAs), focusing on procedural success and complication rates. Materials and Methods: We conducted a retrospective analysis of VAA patients treated at our institution from April 2006 to September 2021. The study reviewed patient demographics, aneurysm characteristics, treatment details, and outcomes, including endoleaks. Results: Among the 23 patients analyzed, splenic artery aneurysms represented 44% of cases. Fifteen patients were treated with balloon-expandable stent grafts (BE SGs), and eight patients were treated with self-expandable stent grafts (SE SGs). For saccular aneurysms, the average neck size was 10.10 ± 8.70 mm in the BE group versus 18.50 ± 3.40 mm in the SE group (*p* = 0.23), with an average sac size of 20.10 ± 18.9 mm in the BE group versus 15.60 ± 12.7 mm in the SE group (*p* = 0.16). The average sac-to-neck ratio was 1.69 ± 2.23 in the BE group versus 1.38 ± 0.33 in the SE group (*p* = 0.63). The BE group exhibited a significantly higher endoleak rate (60%) compared to the SE group (12.5%; *p* = 0.03). Conclusions: While further investigation is needed to fully assess the outcomes of stent graft treatment for VAAs, initial data show a significantly higher endoleak rate with BE SGs compared to SE SGs. The SE SGs may offer better outcomes due to their superior ability to conform to tortuous and mobile visceral arteries.

## 1. Introduction

Visceral artery aneurysms (VAAs), though rare, pose significant clinical risks, with a prevalence estimated between 0.1% to 2% in the general population. These aneurysms primarily affect the splanchnic arteries and may arise from degenerative processes or idiopathic conditions. The advent of advanced imaging technologies has improved the detection of asymptomatic VAAs, but the high risk of rupture—with mortality rates ranging from 8.5% to 100%, depending on the aneurysm’s state and presentation—necessitates timely and effective intervention [1,2,3,4,5].

The criteria for intervention generally include aneurysms larger than 2 cm or those showing signs of growth, due to the significant rupture risk. The selection of treatment modalities is influenced by various factors, including the aneurysm’s size, location, and patient-specific comorbidities, complicating the management approach [2,3,6]. Endovascular techniques have transformed the management landscape of VAAs, offering high technical success with minimal complications, thereby providing a suitable option for high-risk surgical candidates. Among these techniques, stent graft exclusion—which utilizes either self-expandable (SE) or balloon-expandable (BE) stents—remains fundamental. SE stents, typically made from flexible materials like nitinol, are preferred for their ability to conform to the vessel’s natural contours. In contrast, BE stents are chosen for their precise placement capabilities in straighter vascular sections [4,5,6,7,8,9,10,11].

Despite the critical roles of these stents, comparative studies between SE and BE stents in VAA treatment are limited, often anecdotal, or derived from small patient series [6,11,12,13,14,15,16,17,18]. This study seeks to fill this gap by retrospectively analyzing the efficacy, safety, and technical outcomes of SE versus BE stents in the treatment of VAAs at our institution, supported by an extensive review of existing literature and detailed subgroup evaluations. This research aims to provide a detailed comparative analysis, setting a foundation for future studies that might further optimize endovascular strategies for VAA management.

## 2. Materials and Methods

### 2.1. Study Design and Patient Demographics

This retrospective, single-center study analyzed patient records from January 2006 to December 2021. The research adhered to the ethical standards set by the institutional review board, and all participants or their legal representatives provided written informed consent. The inclusion criteria targeted patients with visceral artery aneurysms (VAAs) treated with either self-expandable (SE) or balloon-expandable (BE) stent grafts, ensuring a comprehensive overview of the diverse treatment scenarios and outcomes within this group.

### 2.2. Patient Selection and Preoperative Assessment

Patients were selected based on clinical indications such as imminent risk of rupture identified through imaging, as well as patient-specific factors like pregnancy and portal hypertension [12,13,14,15,16,17,18,19]. The therapeutic approach was guided by aneurysm characteristics, including size (typically over 2 cm), morphology (saccular vs. fusiform), and vascular anatomy (tortuosity, vessel size, proximity to critical branches). This approach balanced vessel-sacrificing and vessel-sparing techniques to optimize outcomes. Visceral artery aneurysms smaller than 2 cm were treated in cases where either the patient’s symptomatic presentation or specific aneurysm characteristics (e.g., rapid growth or saccular morphology) indicated a higher risk of rupture.

### 2.3. Interventional Procedure

The choice of endovascular treatment was determined by the operator based on imaging findings, expediency, hemodynamic status, lesion morphology, systemic or local comorbidities, and anatomical details. The choice between SE and BE stents was based not only on vessel tortuosity but also on the operator’s assessment of the need for precision placement versus flexibility, historical performance data of the stent types in similar anatomical settings, and the patient’s previous interventions. Covered stents (CSs) were the first-line therapy, selected based on the probability of reaching the affected artery with the introducer sheath or guiding catheter, the distance between the lesion and the aorta, and the tortuosity of the affected artery. All procedures were performed under moderate sedation in an angiographic suite by an interventional radiology attending, assisted by a fellow physician, using digital subtraction angiography (DSA). Angiograms were conducted via a trans-femoral approach. After cannulating the parent artery and characterizing the VAA angiographically in multiprojectional and magnified views, the operator made the final treatment choice. Typically, self-expandable stent grafts (SE SGs) were considered for more tortuous vessels, while balloon-expandable stent grafts (BE SGs) were preferred when precise deployment was the priority. Wire access was obtained beyond the planned treatment area. For CS placement, a guiding catheter or vascular sheath was advanced into the parent vessel giving rise to the VAA, and the CS was deployed. To ensure proper sealing post-deployment, angioplasty was performed using appropriately sized balloons. In cases with side branches or bifurcations, prophylactic embolization with vascular coils or plugs was performed at the operator’s discretion to prevent endoleaks. Prophylactic embolization was performed in 12 out of the 23 patients to secure side branches at risk of endoleak. Completion angiography was conducted to confirm the appropriate exclusion of the VAA and continued flow in the parent and desired distal vessel. Post-procedure, patients were administered dual antiplatelet therapy comprising aspirin (100 mg daily) and clopidogrel (75 mg daily) for six months to ensure stent patency.

### 2.4. Imaging Techniques and Data Collection

**Figure 1:**
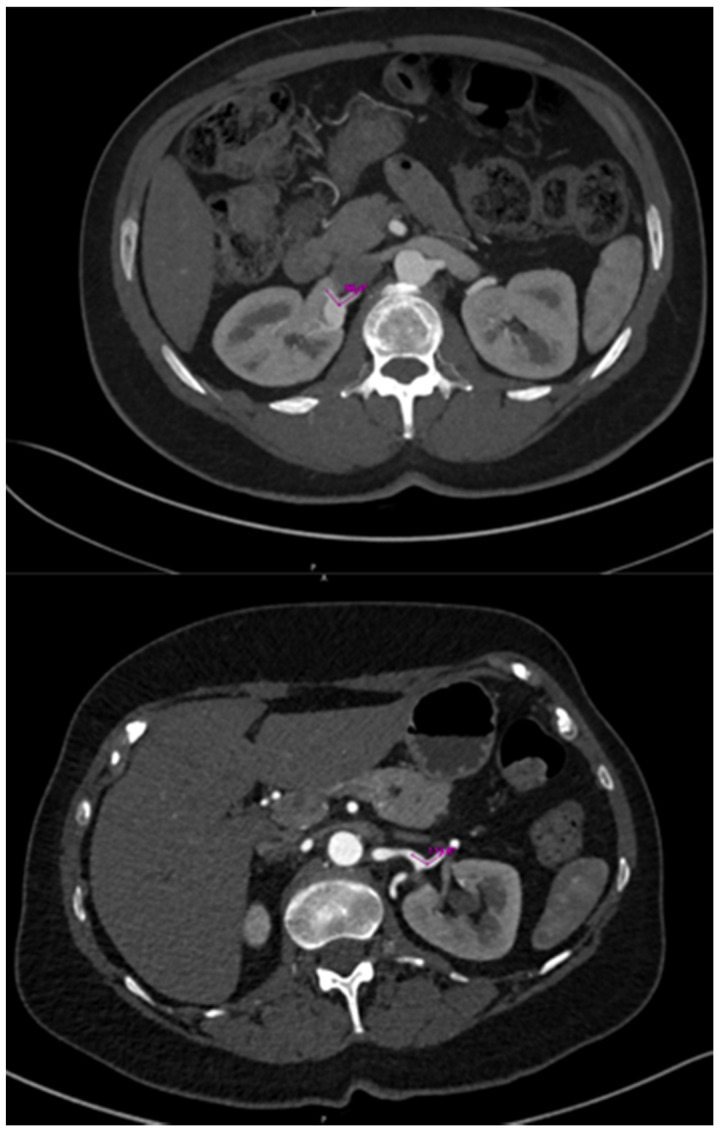
The efferent-to-afferent angle was measured in the axial plane on the most recent cross-sectional imaging prior to intervention.

Extensive pre-interventional imaging was undertaken to measure vessel angles and other critical anatomical features (Figure 1). This data informed procedural planning and stent selection. Comprehensive data collection included aneurysm characteristics, procedural details, post-treatment outcomes such as morbidity, mortality, reintervention rates, complications, and the etiology of the aneurysms (Table 1).

### 2.5. Outcome Measures and Definitions

Technical success was defined as complete angiographic exclusion of the aneurysm without any detectable endoleak post-procedure. Clinical and imaging success included criteria such as aneurysm shrinkage or stabilization, sustained patency of the stent and the parent vessel, absence of organ ischemia, and resolution of symptoms without subsequent aneurysm-related mortality. Primary patency and aneurysm exclusion were verified through follow-up imaging such as computed tomography angiography (CTA) or DSA, ensuring no residual perfusion within the aneurysm sac.

### 2.6. Statistical Analysis

Continuous variables were presented as mean ± standard deviation, and categorical variables as percentages. Long-term outcomes, such as stent-graft patency and re-intervention-free survival, were analyzed using Kaplan–Meier methods with the statistical software IBM SPSS Statistics (Version 20, IBM, Armonk, NY, USA). Associations between qualitative variables were evaluated using Fisher’s exact test, and relationships between quantitative variables were examined using Spearman’s correlation test. A *p*-value of less than 0.05 was considered statistically significant.

## 3. Results

### 3.1. Patient Characteristics

Our study included 23 patients with a mean age of 61 ± 12.2 years, consisting of 13 males and 10 females. The majority, approximately 78%, presented with abdominal pain. Aneurysms were predominantly located in the splenic artery (44%), followed by the hepatic artery (26%) and renal artery (17.5%). The celiac trunk was involved in two cases, and the gastroduodenal and superior mesenteric arteries were used in one case each. Balloon-expandable stent grafts (BE SGs) were used in 15 patients, while self-expandable stent grafts (SE SGs) were chosen for 8 patients based on anatomical considerations and clinical needs. Subgroup analysis based on aneurysm location revealed similar procedural success and complication rates (*p* > 0.05) across all groups, despite anatomical challenges.

### 3.2. Imaging Findings

Pre-intervention imaging showed a mean neck size of 10.10 ± 8.70 mm in the BE group versus 18.50 ± 3.40 mm in the SE group, with no statistical significance (*p* = 0.23). The mean aneurysm sac size was 20.10 ± 18.9 mm for the BE group and 15.60 ± 12.7 mm for the SE group, with the sac-to-neck ratio also showing no significant difference (1.69 ± 2.23 vs. 1.38 ± 0.33, *p* = 0.63).

### 3.3. Clinical Outcomes and Follow-Up

During the follow-up period, stent-graft patency and aneurysm exclusion were evaluated in all patients, although the length of follow-up varied due to the availability of imaging data. Imaging during the follow-up was available for 18 patients at 6 months, 12 patients at 12 months, and 7 patients at 36 months, with a mean follow-up time of 28 months (range, 0–111 months).

Out of the 23 patients, 21 underwent follow-up after the procedure. Imaging assessments provided information on aneurysm exclusion and stent-graft patency, with a mean imaging follow-up time of 31 months for these 21 patients. Seven patients required re-intervention due to endoleak or occluded stent-grafts identified on the follow-up imaging. Clinical and imaging success at 30 days post-procedure was achieved in 11 patients.

When evaluating outcomes based on the type of stent used, both self-expandable (SE) and balloon-expandable (BE) stents achieved a 100% technical success rate. However, the BE group exhibited a significantly higher endoleak rate compared to the SE group. Despite this difference, other complications and overall procedural success rates showed no significant differences between the stent types (*p* > 0.05). Endoleak complications were observed in ten patients, with the BE group experiencing a significantly higher rate of endoleaks at 60%, compared to 12.5% in the SE group (*p* = 0.03). Specifically, the BE group had five Type IA endoleaks, three Type IB, one Type II, and one Type III, whereas the SE group had only a single Type II endoleak. Initial patency rates were 80% for BE stents and 100% for SE stents after 30 days of the procedure (*p* = 0.52).

### 3.4. Long-Term Patency and Survival

**Figure 2:**
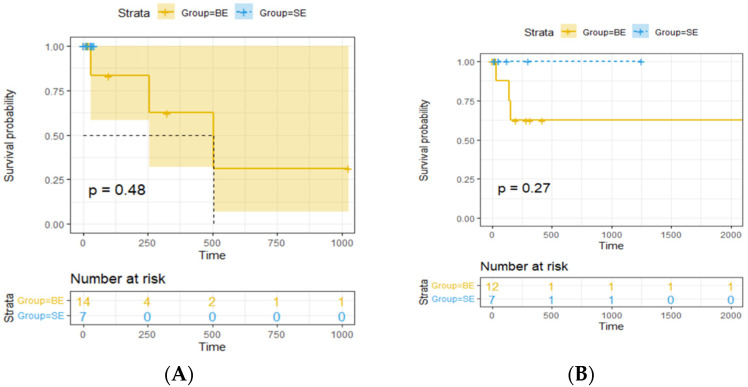

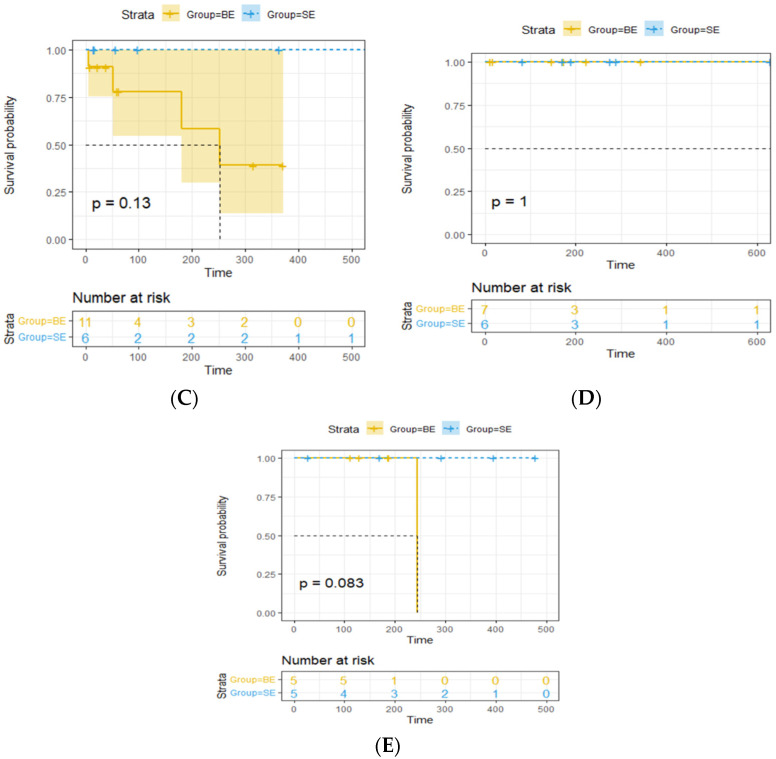
Kaplan–Meier plots comparing the survival rates between the two groups (**A**–**E**).

**Figure 3:**
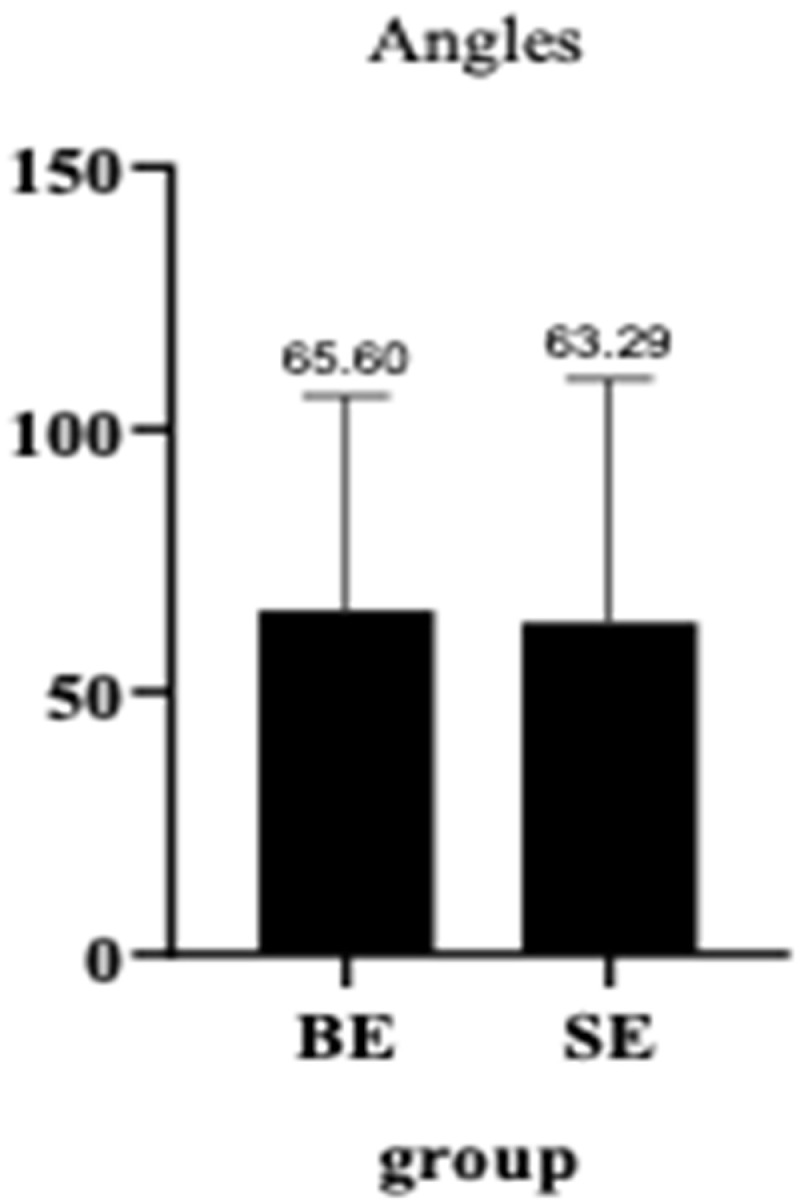
Bar plot comparing the angles between the two groups.

**Figure 4:**
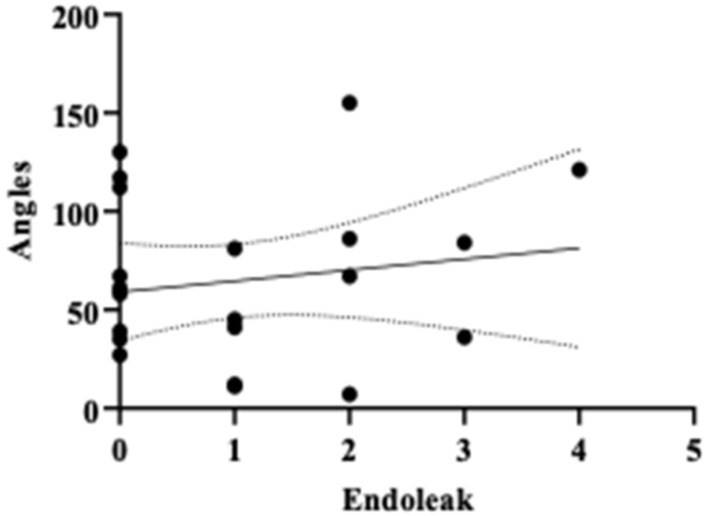
Scatter plot assessing the relationship between angles and endoleak.

**Figure 5:**
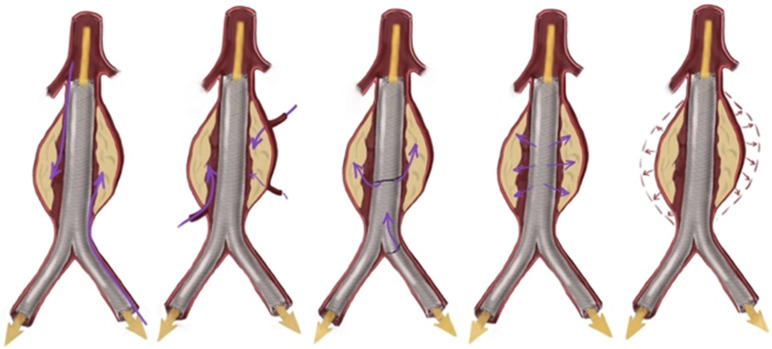
A schematic showing an endoleak classification system. Types of endoleaks are classified according to established criteria: Type Ia represents leakage at the proximal stent graft attachment site, Type Ib at the distal attachment site, Type II results from retrograde flow into the aneurysm sac via collateral vessels, Type III is due to stent graft fabric porosity or structural failure, and Type IV is associated with stent graft material degradation.

**Figure 6:**
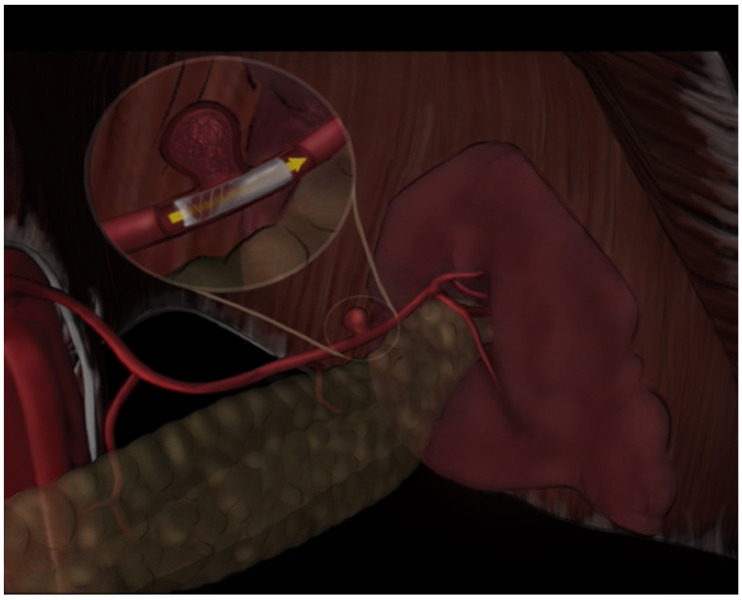
A schematic showing occurrence of endoleaks, one of the most common complications in endovascular procedures, resulting in increased morbidity, mortality, and the need for re-intervention.

**Figure 7:**
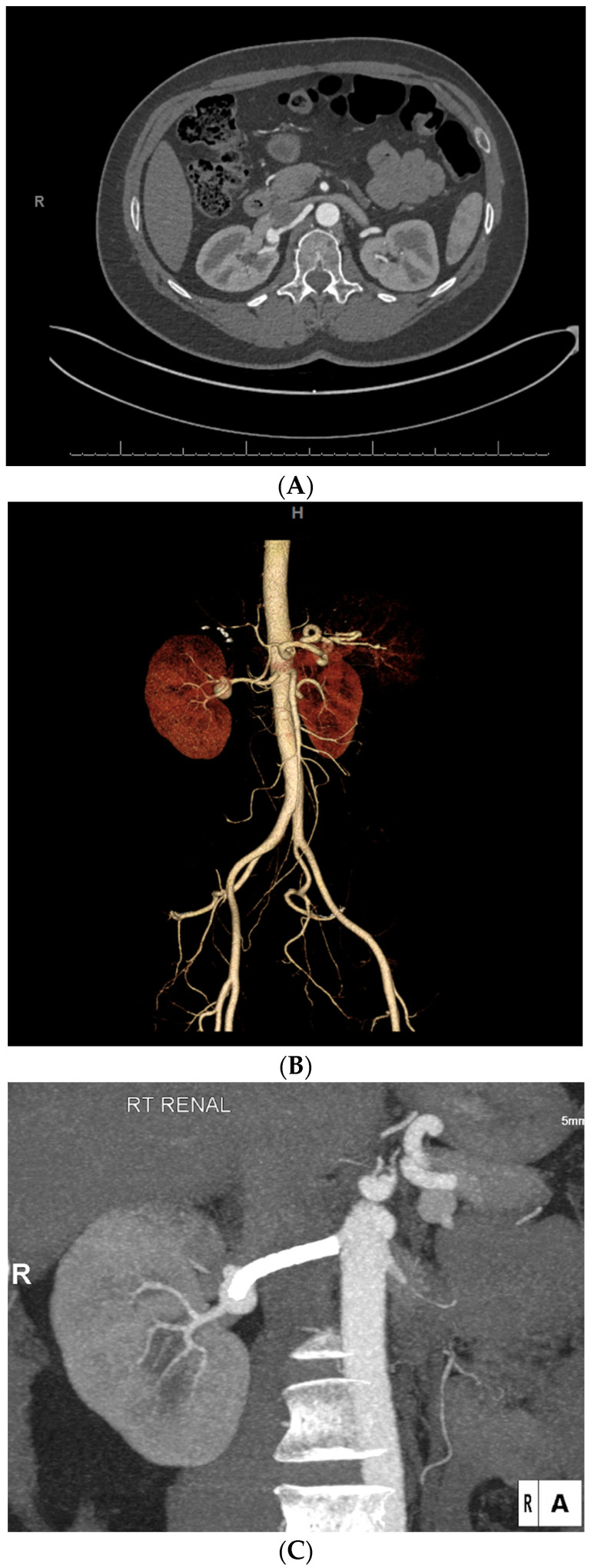
A 57-year-old woman with a significant medical history including gastroparesis, eosinophilic esophagitis with prior dilation in 2020, type 2 diabetes mellitus, multiple sclerosis, anxiety, postoperative nausea and vomiting triggered by narcotics, and multiple medication allergies. She sought evaluation due to an 18 mm right renal aneurysm discovered during a recent CT scan prompted by right flank pain. After consultation, she opted to undergo the recommended surgical intervention for the aneurysm. The computed tomographic angiography of the abdomen and pelvis, both without and with contrast, revealed a saccular aneurysm located at the bifurcation of the right renal artery. The neck size of the aneurysm measures 14 mm, with a maximal diameter of approximately 1.8 cm in the axial plane. After the procedure, follow-up imaging revealed a new stent extending along most of the right renal artery, spanning across an aneurysm located at the renal hilum, which is positioned at a trifurcation. The stent is patent, and the aneurysm remains perfused, measuring approximately 1.7 × 1.5 cm, unchanged from the previous examination (**A**–**C**).

A regression survival model with repeated measures (recurrent survival analysis) demonstrated no statistically significant difference in the time until the occurrence of adverse events between patients receiving BE SGs and those receiving SE SGs, at the 95% confidence level (*p* = 1) (Table 2). Kaplan–Meier curves for stent-graft patency and reintervention-free survival also indicated no significant differences across five separate follow-up periods (Figure 2). The mean efferent-to-afferent vessel angle was 63.29 ± 46.34 degrees in the SE group and 65.00 ± 40.89 degrees in the BE group. A two-sample independent *t*-test revealed no statistically significant difference between the mean angles in the two groups at the 95% confidence level (*p* = 0.907) (Figure 3). The Spearman correlation test, used to examine the relationship between vessel angles and the presence of endoleak, resulted in a correlation coefficient of 0.11, indicating no statistically significant relationship between the two variables (*p* = 0.627) at the 95% confidence level (Table 3, Figure 4, Figure 5, Figure 6 and Figure 7).

## 4. Discussion

The treatment paradigm for visceral artery aneurysms (VAAs) has shifted from traditional surgical approaches to endovascular techniques, with self-expandable (SE) and balloon-expandable (BE) stent grafts (SGs) being prominent options. Endovascular repair with SE or BE SGs allows for aneurysm exclusion while preserving the parent vessel and distal perfusion, theoretically reducing the risk of downstream organ ischemia [11,18,20,21,22]. This approach significantly improves outcomes by decreasing recovery time, hospital stay [1], perioperative morbidity, and 30-day mortality [17,23]. Currently, there is no consensus on the treatment indications for VAAs [11], and image-based monitoring is often recommended for the initial management of small aneurysms, particularly those less than 25 mm in diameter [24].

While endovascular intervention is effective for both symptomatic and asymptomatic patients, asymptomatic lesions have shown lower 30-day mortality compared to symptomatic lesions [25]. This difference may be due to the more severe presentation in symptomatic patients, often with already ruptured aneurysms, compared to asymptomatic patients with intact or smaller aneurysms. Although coil embolization (CS) has shown effectiveness in the VAA treatment, with technical success rates ranging from 84.2% to 98.3% [11,18,20,22], comparative data between SE and BE SGs are limited. Our comparative analysis of SE and BE SGs in managing VAAs has elucidated critical differences in clinical and technical performance. Notably, the lower incidence of endoleaks with the SE stents (12.5%) compared to the BE stents (60%) is significant (*p* = 0.03). This outcome aligns with existing literature, emphasizing the superior adaptability of SE stents to complex vascular architectures, largely due to the flexible properties of nitinol, which enable better conformity to vessel walls [11,18,20,21,22,23,24].

Despite achieving a 100% technical success rate for both stent types over the follow-up period, the higher prevalence of early complications, primarily Type I and Type III endoleaks, in the BE group points to potential issues with their rigidity, especially in anatomically challenging scenarios. The rigidity of BE stents, while beneficial for precise deployment, appears less effective in irregular or highly tortuous vessels [24,25]. This suggests that technical success at deployment does not necessarily equate to optimal clinical outcomes, highlighting a critical area for improvement [26,27,28,29,30,31,32,33,34]. Moreover, our findings did not reveal significant differences in the sac-to-neck ratios between groups, indicating that the geometric compatibility of the stents alone does not reliably predict success in preventing endoleaks. Instead, the material and mechanical properties of the stents emerge as pivotal factors influencing clinical outcomes.

Long-term outcomes, assessed through survival analysis and Kaplan–Meier curves, showed no significant differences in stent-graft patency or reintervention rates between the groups. This suggests that both stent types may stabilize within the vascular system once initial complications are managed, implying their comparable efficacy over the long term. Several anatomical considerations are crucial for ensuring technical success and long-term patency. These include neck length, aneurysm location, and the angulation of the aneurysmal tract, particularly for saccular aneurysms, which were most common in this cohort [2,35]. The tortuosity of the involved arteries plays a significant role and may explain the differences in endoleak complications between BE and SE SG procedures [16,36]. SE SGs, due to their shape memory, are more flexible and conform better to the tortuous visceral arteries. In contrast, BE SGs are relatively rigid, forcing the artery to adapt to the stent’s shape, potentially increasing endoleak rates [22]. Therefore, BE SGs should be considered primarily for VAAs located in short, straight arterial segments or proximal vessels near the aorta.

Another risk factor for the endoleak is the length of the landing zone [37,38]. Adequate proximal and distal landing zones are necessary for proper sealing and aneurysm exclusion [39]. During balloon inflation, the BE SGs and vessels are straightened, but post-deflation the vessel’s tortuosity can lead to decreased seal and increased Type I endoleaks [40]. While a landing zone of 5 mm is generally effective in atherosclerotic lesions, BE SGs require longer zones. Stent oversizing, typically 15–20%, can enhance sealing but must be approached with caution to avoid stent graft folding and subsequent endoleaks [28,41,42,43].

While vessel tortuosity significantly impacts stent deployment and occurrence of the endoleaks, vessel angle remains an important consideration in procedural planning and stent selection. Interestingly, our study found no significant correlation between pre-procedural vessel angles and the occurrence of endoleaks, challenging traditional views that prioritize anatomical considerations in the stent selection [5]. This underscores the need for a more nuanced approach that also considers stent characteristics and deployment strategies during procedural planning. The placement of CS in visceral arteries is also technically challenging due to the severe tortuosity and small caliber of these arteries [9,44]. Utilizing stable carrier systems at the artery origin, hydrophilic guidewires for engaging efferent vessels, or stiff guidewires can facilitate SG advancement during the procedure [22]. This nuanced evaluation underscores the complexity of vascular anatomy and guides the optimization of endovascular treatments for achieving successful outcomes in clinical practice.

Considering the evolving landscape of VAA management, it is pertinent to acknowledge emerging techniques such as flow diversion stents, which offer an alternative strategy and are particularly valuable when preserving branch vessel patency is critical. Flow diversion techniques redirect blood flow away from the aneurysm sac while maintaining the perfusion of vital branches, reflecting evolving strategies that aim to balance effective aneurysm exclusion with minimal disruption to vascular anatomy. Furthermore, discussion of the open surgical approaches remains relevant, especially in cases involving complex or hilar aneurysms where endovascular techniques may pose technical limitations. Surgical intervention continues to play a pivotal role in scenarios requiring direct vessel repair or when endovascular options prove insufficient or impractical. Our study has limitations, including its retrospective design and the small sample size, which may affect the generalizability of the findings. Additionally, being confined to a single institution might introduce biases related to specific interventional practices and patient selection. Future research should expand to multicenter studies to enhance the validity of the results across diverse settings. Also, the absence of long-term follow-up for all participants and a non-randomized design limits our ability to definitively assert the superiority of one stent type over another. Prospective, randomized trials are needed to further delineate the relative benefits and risks of SE and BE stents in VAA management.

## 5. Conclusions

In conclusion, while both SE and BE stents demonstrate high technical success rates, SE stents may offer better clinical outcomes in the management of VAAs, particularly in cases involving complex vascular anatomy. The choice of stent types should be carefully considered, with attention to both the anatomical challenges and the specific properties of the stents. Continued research, especially studies with larger and more diverse populations, is crucial to further elucidate these findings and optimize the treatment strategies for VAA patients.

## Figures and Tables

**Table 1:** Characteristics of included patients.

Patient No	Age	Stent Type	Gender	Location	Size mm	Type of Aneurysm	DOP	Stent Size	Endoleak	Technical Success	Clinical Success	Patent at Latest FU
1	53	BE	F	RAA	17.3	S	1/6/2021, 3/1/2021	6 mm × 39 mm Gore VBX and 6 mm × 19 mm Gore VBX	Endoleak Type IB	No	No	Yes
2	68	BE	F	RAA	28.7	S	9/8/20	5 mm × 22 mm Atrium iCast	No	Yes	Yes	Yes
3	64	BE	M	HAA	48.7	S	2/20/20	7 mm × 59 mm Gore VBX	No	Yes	Yes	Yes
4	63	BE	F	RAA	14.8	S	10/18/19	5 × 22 mm Atrium iCast	No	Yes	Yes	Yes
5	54	BE	F	HAA	19	F	8/2/2018, 8/15/2018, 11/1/2018	6 × 39 mm Gore VBX	Endoleak Type IA	No	No	No
6	59	BE	M	SAA	20	S	8/9/17	8 × 59 mm Gore VBX	No	Yes	Yes	Yes
7	62	BE	M	SAA	17	S	7/7/2017, 1/18/2018	6 × 39 mm Gore VBX and 8 × 39 mm Gore VBX	Endoleak Type IB	No	No	Yes
8	67	BE	M	GDA	77.5	F	12/17/2016, 1/25/2017, 1/26/2017	Procedure 1: 36 × 7 Atrium iCast Procedure 2: 7 mm × 22 Atrium iCast and 7 × 38 Atrium iCast Procedure 3: 8 mm × 50 Viabahn Stent graft and 9 mm × 38 mm Atrium iCast	Endoleak Type III	No	No	Yes
9	52	BE	F	HAA	16	S	2/6/13	7 mm × 22 mm Atrium iCast and 6 mm × 22 mm Atrium iCast and 8 mm × 25 mm Viabahn stent graft	Endoleak Type I A	No	No	Yes
10	71	BE	F	RAA	19.5	S	4/13/2011, 5/29/2020	Procedure 1: 5 × 22 Atrium iCast Procedure 2: 6 × 19 mm Gore VBX	Endoleak Type IA	No	No	Yes
11	79	BE	M	Celiac AA	33.8	S	2/19/09	7 mm × 38 cm Atrium iCast and 8 mm × 38 mm Atrium iCast	No	Yes	Yes	Yes
12	70	BE	F	Celiac AA	36	F	4/26/07	9 mm × 59 mm Atrium iCast	Endoleak Type IB	No	No	Yes
13	64	BE	M	SAA	18.6	S	4/27/16	7 × 22 Atrium iCast (×2)	Endoleak Type IA	No	No	Unclear (limited follow up; splenic artery subsequetly extensively embolized)
14	35	BE	M	SMA	26.1	S	5/5/2017, 5/8/2017	Procedure 1: 7 × 19 mm and 6 × 19 mm Gore VBX Procedure 2: 8 × 50 mm Gore VBX	Endoleak Type IA	No	No	No
15	59	BE	M	SAA	20.7	S	8/9/17	8 mm × 59 mm Gore VBX.	Repeat (No endoleak)	No	No	Yes
16	30	SE	F	SAA	20.4	F	6/4/18	6 mm × 5 cm Viabahn stent graft	Endoleak Type II (resolved)	No	No	Yes
17	70	SE	M	SAA	21.1	S	5/4/18	8 × 50 mm Viabahn stent graft	No	Yes	Yes	Yes
18	81	SE	F	HAA	18.3	F	10/26/16	5 mm × 4 cm Viabahn stent graft and 5 mm × 5 cm Viabahn stent graft	No	Yes	Yes	Yes
19	66	SE	M	HAA	16.2	S	1/1/2016, 1/14/2016	6 × 25 Viabahn stent graft and 6 × 50 Viabahn stent graft	No	No	No	Yes
20	66	SE	M	SAA	24	S	12/13/13	7 mm × 5 cm Viabahn stent graft	No	Yes	Yes	Yes
21	69	SE	M	SAA	18.8	F	2/14/13	6 mm × 5 cm Viabahn stent graft	No	Yes	Yes	Yes
22	58	SE	F	SAA	35.5	F	6/5/08	5 mm × 5 cm Viabahn stent graft	No	Yes	Yes	Yes
23	47	SE	M	HAA	?	F	9/16/06	6 × 40 Viabahn stent graft	No	Yes	Yes	Yes

Abbreviations: DOP, date of procedure; HAA, hepatic artery; RAA, renal artery; SAA, splenic artery; SMA, superior mesenteric artery.

**Table 2:** Comparison of mean angles in SE and BE groups using two-sample independent *t*-test.

	Group	*n*	Mean	Std. Deviation	*p*-Value
Angles	BE	15	65.66	40.89	0.907
	SE	7	63.29	46.34	

In the table, results of two-sample independent *t*-test showed that the difference between the mean angles in the two groups was not significant (*p*-value > 0.05).

**Table 3:** Relationship between angles and endoleak using Spearman test.

		Endoleak
Angles	*n*	22
	Correlation Coefficient	0.110
	*p*-value	0.627

The results of correlation coefficient test showed that there was no statistically significant relationship between angles and endoleak at the 95% confidence level (*p*-value = 0.627).

## Data Availability

The original contributions presented in the study are included in the article, further inquiries can be directed to the corresponding author.
